# Medical assistance in dying for people living with mental disorders: a qualitative thematic review

**DOI:** 10.1186/s12910-023-00971-4

**Published:** 2023-10-24

**Authors:** Caroline Favron-Godbout, Eric Racine

**Affiliations:** Pragmatic Health Ethics Research Unit, Montreal Clinical Research Institute, 110 av. des Pins O, Montreal, QC H2W 1R7 Canada

**Keywords:** Ethics, Bioethics, Medical assistance in dying, Euthanasia, Physician assisted Suicide, Mental disorders

## Abstract

**Background:**

Medical assistance in dying (MAiD) sparks debate in several countries, some of which allow or plan to allow MAiD where a mental disorder is the sole underlying medical condition (MAiD-MD). Since MAiD-MD is becoming permissible in a growing number of jurisdictions, there is a need to better understand the moral concerns related to this option. Gaining a better understanding of the moral concerns at stake is a first step towards identifying ways of addressing them so that MAiD-MD can be successfully introduced and implemented, where legislations allow it.

**Methods:**

Thus, this article aims (1) to better understand the moral concerns regarding MAiD-MD, and (2) to identify potential solutions to promote stakeholders’ well-being. A qualitative thematic review was undertaken, which used systematic keyword-driven search and thematic analysis of content. Seventy-four publications met the inclusion criteria.

**Results:**

Various moral concerns and proposed solutions were identified and are related to how MAiD-MD is introduced in 5 contexts: (1) *Societal context*, (2) *Healthcare system*, (3) *Continuum of care*, (4) *Discussions on the option of MAiD-MD*, (5) *MAiD-MD practices*. We propose this classification of the identified moral concerns because it helps to better understand the various facets of discomfort experienced with MAiD-MD. In so doing, it also directs the various actions to be taken to alleviate these discomforts and promote the well-being of stakeholders.

**Conclusion:**

The assessment of MAiD-MD applications, which is part of the context of MAiD-MD practices, emerges as the most widespread source of concern. Addressing the moral concerns arising in the five contexts identified could help ease concerns regarding the assessment of MAiD-MD.

**Supplementary Information:**

The online version contains supplementary material available at 10.1186/s12910-023-00971-4.

## Background

Medical assistance in dying (MAiD), which encompasses euthanasia and physician-assisted suicide [1], raises debate in several countries. Belgium, The Netherlands, Luxembourg, and Switzerland have allowed people living with mental disorders to access some forms of MAiD for years [2]. More recently, Spain has passed a MAiD law making people living with mental disorders eligible for MAiD under certain conditions [3]. Canada has decriminalized MAiD for physical conditions and plans to allow MAiD when a mental disorder is the sole underlying medical condition (MAiD-MD) [4]. The subject of MAiD-MD is a delicate and controversial one, which has given rise to a great deal of international reflection, leading to the development of a rich literature on the subject. Hence, the literature on MAiD-MD is extensive, and research emerging from countries that allow this practice is complemented by contributions from countries that do not. An important part of the reflections and work carried out on the subject regards the question of whether people living with mental disorders should be eligible for MAiD or not. Literature reviews on the arguments in favor and against MAiD-MD have notably been carried out by Nicolini et al. (2020) and Grassi et al. (2022) [5, 6]. The ethical acceptability of MAiD-MD practices is a polarizing issue, which can limit the exploration of nuances in positions and impede the mutual understanding of people with different perspectives on this question. We believe that a promising notion for exploring these nuances is that of moral concerns, as they may provide common ground for discussion between those in favor and those against MAiD-MD. Indeed, moral concerns may be part of the reasons why some people are opposed to MAiD-MD, but also of the drawbacks that those in favor of MAiD-MD feel are important to address if the practice is to be acceptable. For example, moral concerns about the conciliation of MAiD-MD practices and suicide prevention practices may lead some people to oppose MAiD-MD, just as it may qualify the position of those in favor of MAiD-MD (e.g., being in favor on condition that requests for MAiD-MD are not the result of suicidal impulses).

Considering that MAiD-MD is becoming permissible in a growing number of jurisdictions, there is a need to better understand the moral concerns related to this practice. Gaining this understanding is a first step towards identifying ways of addressing them so that MAiD-MD can be successfully introduced and implemented, where legislations allow it. Thus, this article aims (1) to better understand the moral concerns regarding MAiD-MD, and (2) to highlight potential solutions that have been suggested by others to promote stakeholders’ well-being (namely people living with mental disorders, their relatives, and their healthcare professionals). Hence, although highly relevant, moral concerns relating to not allowing MAiD for people with psychiatric suffering are not covered by this literature review.

## Methods

**Fig. 1:**
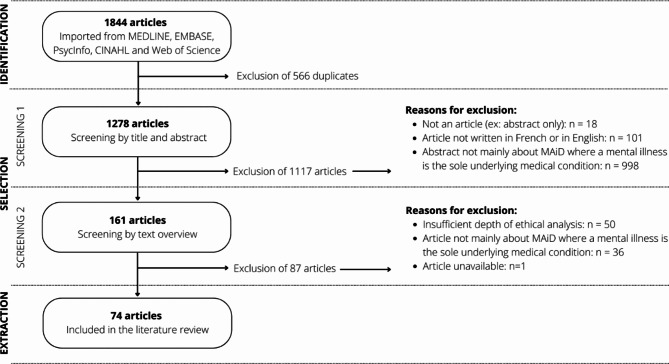
PRISMA diagram illustrating the systematic article selection process

This qualitative thematic review relied on systematic literature searches in addition to screening and structured thematic content extraction strategy, inspired by Arksey & O’Malley (2005) and by Levac & al. (2010), as well as by more structured forms of thematic literature reviews [7–12]. Figure 1 illustrates the article selection process in the form of a PRISMA-type diagram.

Identifying the research question: The following questions guided the review: What moral concerns are reported regarding medical assistance in dying where a mental disorder is the sole underlying medical condition? What possible solutions are proposed to address these concerns?

Identifying relevant studies: The literature searches were developed around three concepts: moral concerns, medical assistance in dying, and mental disorders. The search strategy was validated by a librarian from Université de Montréal’s School of Public Health and put forward MeSH terms (Medical Subject Headings) and keywords for each concept, which were applied to the title, abstract, and keyword headings fields. Moral concerns were identified using the “ethics” and “morals” MeSH terms, with the equation (Moral* or bioethic* or ethic*); medical assistance in dying was targeted using the MeSH terms “suicide, assisted”, and “euthanasia, active, voluntary”, with the equation ((Assist* or “medical aid”) ADJ2 (dying or dead or death or suicide or die)) or euthanas*; mental disorders were targeted with the “mental disorders” MeSH term, with the equation mental disorder* or mental health or mental illness* or psychiatric disorder* or psychiatric illness* or psychiatric disease* or behavior disorder* or bipolar* or depress* or psychos* or psychot* or schizoaffect* or schizphren* or cyclothym* or anxiety* or obsessive-compulsive disorder* or post-traumatic stress* or dissociat* disorder* or personality disorder* or eating disorder* or social phobia or substance-related disorder*. The literature search was conducted on July 13, 2022, in the MEDLINE (n = 641), EMBASE (n = 509), CINAHL (n = 170), PsycInfo (n = 268) and Web of Science (n = 256) databases, and the identified articles were imported into the Covidence software (N = 1278).

Study selection: Article selection was conducted in two phases: (1) by the title and the abstract, (2) by the content overview. The inclusion criteria for the first screening phase were: that it is an article and not a poster or conference abstract; that the article is written in French or in English (languages mastered by the authors); that the abstract of the article focuses on MAiD-MD. This first screening phase allowed for 161 articles to move to the second screening phase. Articles were excluded if MAiD-MD was not central to the abstract; if they dealt with MAiD in a context other than mental disorders (e.g., MAiD only, MAiD for physical conditions or for life fatigue, MAiD in the context of neurocognitive or neurodevelopmental disorders, MAiD for minors and newborns); if they dealt with suicide only or palliative care; if they dealt with involuntary euthanasia (e.g., Nazi era); or if they addressed moral concerns unrelated to MAiD-MD (e.g., veterinary euthanasia, health ethics in general). The inclusion criteria for the second phase were: that the article focuses on MAiD-MD (e.g., an article about MAiD but with more than minimal content about MAiD-MD); that the article offers substantial qualitative analysis either by their methods (qualitative design) or by qualitatively appraising quantitative data (quantitative design); that the article mentions moral concerns related to MAiD-MD (e.g., an article about the eligibility criteria for MAiD-MD and discussing related moral concerns). This second screening phase allowed for the inclusion of 74 articles. Articles were excluded if MAiD-MD was a minor portion of the article (e.g., if only a brief paragraph alluded to MAiD-MD); if the qualitative contribution of the article was limited or absent (e.g., survey results presented with little interpretative analysis); or if the content was purely clinical. Inclusion criteria were piloted and established by the first author and a research assistant, who independently reviewed each article, and were validated by the second author. Disagreements were resolved through dialogue and contributed to the refinement of inclusion criteria.

Charting the data: The content extraction was carried out by the first author to identify moral concerns emerging from the literature as well as possible solutions to address them. A moral concern was identified when discomfort, distress, uncertainty, or a dilemma as to the best way to act was identified [13] or if a described situation impeded stakeholders’ well-being [14]. Articles were classified as (1) theoretical article; (2) perspective, opinion, response, or comment; (3) review; (4) empirical study; (5) case review or case series; (6) chapter.

Collating, summarizing, and reporting the results: A thematic analysis allowed us to identify moral concerns and to group them into five contexts of emergence, as illustrated in Additional file 1. We have also identified potential solutions proposed in the literature and associated them with the context of emergence to which they correspond. Narrative summaries citing illustrative studies are presented to highlight these moral concerns and proposed solutions. Salient constructs, which recur in several contexts, have been identified in the discussion.

Consultation: Following Levac et al. (2010)’s steps for realizing a scoping review, this literature review included a consultation phase with a group of key stakeholders, including people living with a mental disorder, to complement the literature review [11, 12]. The group’s comments and experiential knowledge were considered to improve the formulation of some of the identified moral concerns and to shed better light on some of them.

## Results

The keyword-driven searches yielded 74 publications corresponding to the inclusion criteria, of which 18/74 came from jurisdictions currently allowing MAiD-MD (Belgium, The Netherlands, Switzerland), 22/74 came from a country planning to expand its legislation (Canada), 19/74 were from places where MAiD-MD is not considered (USA, Africa, Australia, other countries in Europe), and 14/74 were combined international perspectives. Among these publications, 29/74 were theoretical articles; 28/74 were comments or perspectives; 7/74 were reviews; 4/74 were empirical studies; 4/74 were case studies; and 2/74 were chapters.

**Fig. 2:**
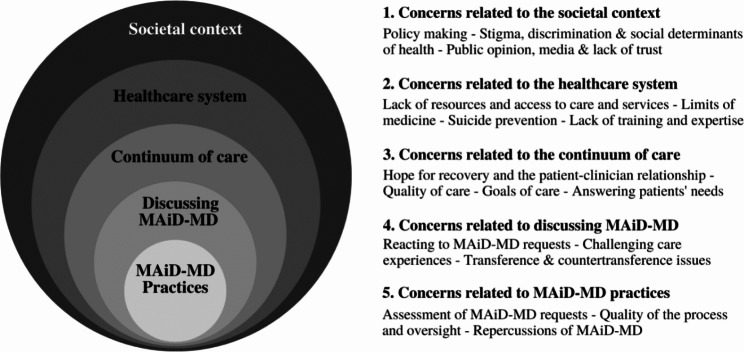
Five contexts of emergence of moral concerns related to MAiD-MD

This qualitative thematic review allowed us to identify various moral concerns and possible solutions, which are related to how MAiD-MD is introduced into the following 5 contexts of emergence: (1) *societal context*, (2) *healthcare system*, (3) *continuum of care*, (4) *discussions on the option of MAiD-MD*, (5) *MAiD-MD practices*. These contexts were identified iteratively during data extraction and are interrelated as shown in Fig. 2.

## Societal context

### Moral concerns related to the societal context

#### Policy making and tension between inclusion and protection

In the societal context, the primary moral concern surrounding MAiD-MD relates to developing laws, policies, and regulations. On the one hand, the need to protect people living with mental disorders is highlighted, given their possible vulnerability [15–17]. On the other hand, excluding these people from eligibility for MAiD-MD is seen as an obstacle to their right to die with dignity [18]. Laws governing MAiD try to take this tension into account. However, concerns remain about the societal responsibility to help these people live with their condition before considering helping them to die [18, 19]. Although MAiD-MD can bring some peace of mind [18, 20, 21] by offering an exit option to people who fear that their suffering will be perpetual [22], concerns have been expressed about the message sent by the expansion of MAiD eligibility to potentially vulnerable people [23], who may be struggling to find meaning to their lives [24]. The fear that the gradual expansion of the laws governing MAiD could lead to abuses is also noted [25, 26].

#### Stigma, discrimination, and social determinants of health

The introduction of MAiD-MD raises fears of increased stigmatization and discrimination about mental disorders and the fear that problems related to social determinants of health may lead some people to consider seeking MAiD-MD. Discrimination can be experienced by people with mental disorders on a daily basis, both by the people around them [27], by the authorities [28], by the medical profession [17], or by public policies that exclude them or fail to consider them adequately [16, 29–32]. Experiences of stigma have been reported as contributing more to the intolerability of suffering among people with mental disorders than the symptoms of the disorder [33]. Some socially disadvantaged people might consider MAiD-MD to escape the suffering and stressors caused by their reality [6], such as social isolation, homelessness, poverty, inadequate housing, and unemployment [33, 34]. Thus, people could ask for MAiD-MD to escape life circumstances that are in principle remediable (e.g., with policy and decisions regarding resource allocation [35]).

#### Public opinion, media and lack of trust

Some authors fear MAiD-MD may lead to or increase the erosion of patient and public trust in psychiatry [25, 36] by reinforcing the belief that nothing can be done to help people living with mental disorders [37]. The growing media coverage of controversial examples of MAiD-MD can amplify this lack of trust [37] by opening a space of doubt and fear in the collective imagination [38] and by influencing policymaking. Finally, a history of mistreatment towards people with mental disorders generates a taboo around MAiD-MD [39].

### Identified needs and proposed solutions to societal context issues

A critical need is to help people live with their mental disorders before considering helping them die, which requires improving social support [40], reducing stigma [41], adapting work environments to mental health needs, and fostering a sense of community belonging [42]. It is essential to examine the legislative developments surrounding MAiD-MD [43] to ensure that the suffering of people living with mental disorders is not minimized [44] and to promote their empowerment in their care decisions as well as in their life trajectories, which may or may not include a request for MAiD-MD [38].

## Healthcare system

### Moral concerns related to the healthcare system

#### Lack of resources and access to care and services

Human, financial, and material resource shortages [34, 45, 46] are acute in mental health care and services. In this context, adding a more rigorous assessment process for MAiD-MD requests, including various safeguards, could impose an additional burden on an already struggling system [28, 45]. Furthermore, access to adequate and patient-centered mental health care is essential to alleviate suffering. However, many people living with mental disorders face significant delays in accessing care and services [46] or do not have access to treatments and services adapted to their condition at all [43]. MAiD-MD could thus be considered an alternative to care, which is deemed problematic [19, 47]. Although this possibility may suggest that it is preferable not to allow MAiD-MD in a context where resources and access to resources are lacking, preventing people in unbearable suffering from accessing MAiD-MD on the pretext that the healthcare system must be improved beforehand is to condemn prima facie eligible people to excruciating suffering [34].

#### Limits of medicine

The fact that, despite decades of research, medicine remains an imprecise discipline that cannot answer every problem is difficult to accept for some healthcare professionals [48]. It can make them reluctant to conclude that a mental disorder is without prospect of recovery; hence, to consider a MAiD-MD request ineligible. In some cases, the lack of effectiveness of mental health treatments [49] can make healthcare professionals feel powerless or dread situations where they cannot help certain people [41]. Furthermore, when quality mental health care fails to help patients, they may be confined to intolerable suffering unless MAiD-MD is considered [48]. The fact that the causes of mental disorders are often misunderstood limits the chances that effective treatments adapted to the patient’s needs will be available [50, 51].

#### Suicide prevention

The main concern surrounding MAiD-MD and suicide prevention is the idea that MAiD-MD is a way of helping some people commit suicide [49, 52]. Therefore, MAiD-MD seems complicated to reconcile with suicide prevention [53, 54], with suicide prevention being a well-established societal responsibility [47]. Some healthcare professionals perceive MAiD-MD as a way to act on suicidal ideation, resulting in the death of people who would not otherwise have committed this act [52]. Thus perceived, MAiD-MD runs counter to the commitment of healthcare professionals to preserve life by preventing suicide [47]. However, data from a recent empirical study reported that people living with mental disorders have no difficulty distinguishing their previous suicidal states from their state when they requested MAiD-MD [20]. This suggests that the reasons leading a person to seek MAiD-MD may differ significantly from those leading them to commit suicide.

#### Lack of training and expertise

A lack of training and expertise from healthcare professionals could impede the introduction of MAiD-MD in the healthcare system [55, 56]. Some healthcare professionals feel ill-prepared to follow the law, failing to know how it applies to patients [56] and how to apply it without discrimination [33] (e.g., without being influenced by biases against mental disorders). Others feel a lack of preparation to receive MAiD-MD requests and to get involved in MAiD-MD [55, 56], either due to a lack of communication skills [57], lack of knowledge of mental disorders [33, 58] or lack of reflexivity and ethical deliberation skills [56].

### Identified needs and proposed solutions to healthcare system issues

Among the proposed solutions, having better-resourced mental health services is necessary [16, 40]. The need to show humility in the face of the limits of psychiatry in relieving mental suffering [24] is reported, and the need to improve access to mental health care and services is also expressed [34, 42, 45, 57]. The importance of raising awareness of suicide risk and its management is mentioned too [37]. In addition, a glaring need is felt among healthcare professionals for training [42, 56] and guidelines considering the complexity encountered in practice [59], and both healthcare professionals, MAiD-MD applicants, and their relatives would benefit from these resources being developed.

## Continuum of care

### Moral concerns related to the continuum of care

#### Hope for recovery and the patient-clinician relationship

The introduction of MAiD-MD into the continuum of care can affect the therapeutic relationship [17, 48, 59], which is closely correlated with patients’ hope for improvement [37], a key facilitator of recovery [23, 52]. Healthcare professionals’ involvement or support of MAiD-MD could also affect the resilience of patients [60], the therapeutic relationship [26, 48, 60] and potentially influence the patient to lose hope that their situation will improve if they have the impression that the healthcare professional is abandoning the therapeutic project [23, 37]. Such loss of hope can give rise to a self-fulfilling prophecy [23, 37, 51] by reinforcing the patient’s sense of hopelessness and compromising their potential for recovery [51]. Conversely, some authors point out that acknowledging the irremediability of a person’s condition can promote hope, empathy and engagement and thus promote recovery [33], making that person feel genuinely considered [37].

#### Quality of care

Another primary concern surrounds the quality and continuity of care [19], and adding MAiD-MD as an option exacerbates this concern [23, 54]. The fear that MAiD-MD might hinder the improvement of care practices, social support, and resources to help people live with their mental disorders has been expressed [37, 41]. It is problematic that a person could request MAiD-MD without first accessing care and services adapted to their situation [61, 62]. For example, Mehlum et al. (2020) mentioned doubting whether people living with borderline personality disorder are offered evidence-based personality disorder-specific treatments. This suggests that people living with mental disorders may seek medical assistance in dying while not receiving the best treatments for their condition [63]. A Dutch empirical study reported that some people who requested MAiD-MD felt they were being refused some therapeutic options because their mental healthcare professional deemed treatment incompatible with a wish for MAiD-MD [20]. A lack of holistic care for people living with mental disorders has also been documented: psychosocial dimensions and interactional factors are often overlooked compared to biological dimensions [23]. Being regularly confronted with patients with suicidal ideation, healthcare professionals can involuntarily adopt paternalistic attitudes towards these people [64], which affects their quality of care.

#### Goals of care

MAiD-MD may seem irreconcilable with the valuing of recovery-oriented approaches in mental health care [65], and some fear that its introduction into the continuum of care will cause a paradigm shift from a goal of improving quality of life towards a goal of assessing eligibility for MAiD-MD [28]. Helping people live with their mental disorders should precede MAiD-MD [66]. Still, the goals of care should also be tailored to each person’s specific needs, preferences, and life goals, including the right to refuse treatments, and the need to recognize when interventions become futile [2, 32, 61, 62, 64, 67]. Healthcare professionals may find themselves uncertain about how best to act in the face of treatment refusals, believing that these refusals can lead to preventable deaths [4] and considering that alleviating suffering through MAiD-MD takes time away from patients, limiting opportunities for healthcare professionals to provide significant therapeutic benefits [38]. Determining the futility of interventions is a difficult value judgment to make for both healthcare professionals and patients [32, 64].

#### Answering patients’ needs

Among the unmet needs of people living with mental disorders are those related to care and services (notably a lack of symptom relief), but also those related to daily needs (notably the need to have satisfying relationships) and existential needs (especially the difficulty of finding meaning to life) [16, 20, 68]. The lack of response to these needs can prevent them from having a satisfactory quality of life [31]. This lack of help in living with mental disorders may lead some people to conclude that MAiD-MD is the only option susceptible to alleviate their suffering. Although MAiD-MD may be an option adapted to the needs of some people living with mental disorders, they must have prior access to options likely to help them live with their condition, knowing that they may decide not to choose these options. These options may include, but are not limited to, healthcare, social services, adapted housing and community support.

### Identified needs and proposed solutions to continuum of care issues

Quality care involves adopting a non-paternalistic attitude towards people living with mental disorders [31, 69]. Reasonable therapeutic alternatives should be tried before considering MAiD-MD [18, 22] while considering the patient’s values. Thus, a change in psychotherapeutic or pharmacological strategy or a change in physician or care setting should first be considered before concluding that no options are left to help the person. For some, the mere possibility of having access to MAiD-MD could be enough to lighten the burden of their mental disorder without feeling the need to take advantage of it [20, 21]. It is essential to cultivate the therapeutic relationship, to maintain open communication, and to offer support to address existential questions [70]. Recognizing and considering their moral convictions may help healthcare professionals be more mindful about discussing MAiD-MD with patients [28]. Communication should be patient-friendly, and the uncertainty related to mental disorders explicitly recognized [71], choosing words so as not to undermine hope (e.g., avoiding saying that nothing more can be done to help the person [72]). Some underline the attention that palliative psychiatry should receive when the mental disorder has reached a certain chronicity, as it could help alleviate some suffering of mental disorders [37, 41, 70]. These avenues seem promising for helping people living with mental disorders lead meaningful lives and maintain a positive view of themselves [62]. Two-track approaches to MAiD-MD, which maintain recovery-oriented care in parallel with the assessment of MAiD-MD requests, are also reportedly promising [40, 69].

## Discussions on the option of MAiD-MD^1^

### Moral concerns related to discussing the option of MAiD-MD

#### Reacting to MAiD-MD requests

Since MAiD-MD is a morally sensitive practice with which not everyone agrees, it can be difficult for healthcare professionals to receive requests for MAiD-MD, to respond to them in a way that suits the patient’s needs, to involve the family or not, and to adapt the treatment goals. It can also be challenging for family members to accept that their loved one intends to request or has requested MAiD-MD [24]. Perception conflicts can arise between patients and healthcare professionals [71, 73], whose training focuses mainly on preserving life, particularly when MAiD-MD is not considered a last resort because treatment options remain to be tried [22]. The way of receiving and responding to the request for MAiD-MD can be tinged with paternalism aimed at imposing healthcare professionals’ values on patients [48] or reveal a lack of empathy or sensitivity to their situation [39], which can increase the distress of applicants. A person who requests MAiD-MD and is simply told that they are not eligible or that they are not there yet may feel frustration [20], even distress. MAiD-MD requests place a significant emotional burden on the healthcare professionals who receive and assess them [24, 55], particularly if they are conscientious objectors [45] or if these requests make them feel powerless to protect people with mental disorders [41, 53]. Conscientious objectors can oppose MAiD-MD without ignoring the suffering at stake: assuming the opposite is a mistake [74]. The experience of being overburdened by the number or the weight of the requests also leads some healthcare professionals to refrain from participating in MAiD [55]. Moreover, having a loved one die from MAiD-MD can be distressing for relatives [55], who may need to reconcile the hope that the patient will recover with the support they want to provide [6]. Although the involvement of relatives can be beneficial to better understand the situation of the person requesting MAiD-MD, confidentiality issues also arise when it comes to whether or not to involve relatives in the discussions surrounding MAiD-MD [45, 75].

#### Challenging care experiences influencing discussions

Many people with mental disorders have difficult care experiences. These people are sometimes met without compassion or even with exasperation, which can affect their self-esteem and increase their marginalization [19]. Mental suffering can be difficult to understand [24, 48] and tends to be less recognized than the suffering arising from physical illnesses [15, 21, 39]. People living with mental disorders are often not taken seriously [20, 48] unless they have physical symptoms or somatic manifestations. Even that is not always enough to make them be taken seriously. They may then feel helpless, which may lead them to seek MAiD-MD. This request can be well considered [42], but it can also be a cry for help in order to be seen and heard [20, 70], or a request for communication [63]. The moral, cultural, or professional biases of healthcare professionals can alter the quality of the care experience [37]. Thus, biases against psychiatric distress can lead healthcare professionals to conclude that the person’s suffering is exaggerated and only in their head [32].

#### Transference and countertransference issues

MAiD-MD can generate issues of transference and countertransference, in particular given the enduring relationship that some patients have with their healthcare professional [2, 28, 53, 59, 60, 71, 76]. A healthcare professional could involuntarily be overinvested [41] or identify too much with the patient’s situation [2, 46] and show them an excess of empathy because they fear the state of deterioration, suffering, and loneliness experienced by the patient. This transposition of the healthcare professional’s emotions onto the situation would constitute countertransference. A healthcare professional could share the patient’s demoralization [57] from lack of being able to relieve their suffering [28]: this transposition of the patient’s despair onto the healthcare professional would constitute transference. Transference and countertransference often come together and are usually unconscious, but they can lead healthcare professionals to facilitate access to MAiD-MD, or to respond to a MAiD-MD request with hostility, which could subsequently make a patient reluctant to share their suicidal thoughts, push them to act on those suicidal thoughts, or lead them to change healthcare professionals, thus harming the continuity of care [28]. The ability to bear the suffering reported by patients while containing their feeling of helplessness is a major personal and relational challenge for healthcare professionals, who are often ill-prepared for this type of communication [57]. Identifying their own feelings of countertransference is also difficult for healthcare professionals [59], who may lack perspective of their situation.

### Identified needs and proposed solutions related to discussing MAiD-MD

The fundamental recommendation concerning the management of MAiD-MD requests is related to the way of receiving and responding to them. Whether the applicant is eligible or not, requests for MAiD-MD should be welcomed with openness and empathy, and should be considered seriously [70]. These requests can be received first as a demand for human connection and professional expertise to relieve suffering [70], but they must also be considered as real requests for assistance in dying to which it is important to respond in a meaningful way [42]. Feeling that their suffering is acknowledged and having the opportunity to talk about their request for MAiD-MD can ease the suffering of people living with mental disorders, give them hope, build trust in the therapeutic relationship, and lead them to consider new therapeutic avenues, or decrease their desire to die [20, 58]. This requires facilitating communication through deliberation and considering the important values of all stakeholders [65, 70] as well as the needs of the MAiD-MD applicant. The issues of transference and countertransference should be addressed through professional consultations where the observations, feelings, and motivations of healthcare professionals would be shared with the consulted colleagues to gain better insight into possible situations of countertransference [71]. However, this might not always be sufficient [59].

## MAiD-MD practices

### Moral concerns related to MAiD-MD practices and their repercussions

#### Assessment of MAiD-MD requests

The assessment of requests is the dimension of MAiD-MD that has received the most attention in the literature. The underlying moral concerns are manifold and relate to the complexity of the assessment of MAiD-MD requests [56, 58]; as well as to the interpretation and application of eligibility criteria [6, 56], mainly in relation to capacity [29, 77, 78], to the intolerability of suffering [17, 56, 64, 70], and to the irremediability of the condition [47, 51]. The vagueness of the concepts of incurability and irremediability, as well as the complexity of assessing the applicant’s level of appreciation of their situation, have also been highlighted [45, 55, 77, 79]. With respect to capacity to consent, many authors are concerned that requests for MAiD-MD can be influenced by the mental disorder and that the desire to die can be a symptom of the disorder, or that the request for MAiD-MD can stem from external pressure [6, 41, 45], which would invalidate the voluntary nature of the request [56, 80]. When remediable external factors are driving the request, the concern that MAiD-MD could become a permanent solution to a temporary problem is palpable [22, 81].

Cognitive distortions resulting from the disorder could invalidate the informed nature of the requests [47], but this is not always the case [44, 79]. The lack of stability of the desire to die, understood as a lack of continuity in the desire to obtain MAiD-MD, emerges as a concern [2]. The fluctuating nature of capacity also complicates its assessment [27, 48]. The question of the intolerability of suffering highlights the subjective nature of this assessment by both the applicant and the healthcare professional [44, 58]. Knowing that the perceived acceptability of an intervention varies across patients [27], it can be difficult for healthcare professionals to confirm that a person’s suffering is intolerable [46]. Attempting to objectively assess suffering can also lead to insensitivity towards a person’s unique experiences of states they consider intolerable [49]. In terms of the irremediability of the condition, the diagnostic and prognostic uncertainty complicates the assessment of eligibility for MAiD-MD [82], in particular because the possibility that a future intervention will succeed in alleviating the suffering still exists [38], but also because refusing treatment can add a layer of complexity to the assessment of irremediability [5]. Rooney et al. (2018) point out, however, that it is epistemically impossible to know with certainty that a person will never recover, and that to require such certainty in assessment condemns some people to suffer without considering the reality of their experiences [16].

#### Quality of the process and oversight

The interpretive leeway enjoyed by healthcare professionals complicates the task of ensuring the quality, rigor and consistency of the MAiD-MD request assessment process [2, 19, 28, 37]. The concern that some healthcare professionals could feel pressured to acquiesce to a request for MAiD-MD because it seems the least harmful avenue for a patient demonstrating chronic suicidality is also implied [22]. In addition, the subjectivity of the assessment increases the risk that it – or the discussions it relies on – contains biases, particularly in relation to the clinical experience, beliefs and values of healthcare professionals [28], or because of existing prejudices against mental disorders (e.g., assuming that all people living with mental disorders lack decision making capacity) [32, 43, 61]. Although collaborative work and discussions between healthcare professionals can limit the impact of biases on the assessment process [71], some assessing healthcare professionals seem reluctant to involve other healthcare professionals who do not bear decision-making responsibility (e.g., members of the healthcare team who know the patient well) [56]. The length and complexity of the assessment process makes some healthcare professionals uncomfortable about having to refuse a request for MAiD-MD [55], or even leads them not to get involved in this practice [46]. Concerns about appropriate safeguards have been raised, including the lack of referral to a psychiatrist [37]; the use of a third party that can impose an arbitrarily high capacity threshold while neglecting the context behind the MAiD-MD request [48]; or the imposition of a longer minimum period between the request and MAiD-MD implementation [48]. Freeland et al. (2022) point out that where some believe that the nature of mental disorders warrants additional safeguards, others consider the imposition of specific measures to be discriminatory [45]. Questions are also raised regarding the quality of regulatory oversight processes for MAiD-MD [37, 75] and the best type of oversight process to implement retrospectively or prospectively [45].

#### Repercussions of MAiD-MD

Concerns related to the repercussions of MAiD-MD include the risk of error in the assessment of requests, the burden and moral distress that can accompany this practice, as well as the possibility that the patients feel left to fend for themselves or are pushed towards suicide. Errors in assessment could result in some patients being incorrectly deemed eligible, and vice versa [19, 47]. They could also put healthcare professionals at risk of sanctions, and some point to a need to protect healthcare professionals in this sense [56]. The burden of bearing the decision can be heavy for healthcare professionals [6]. Some of them may experience moral distress if they think the eligibility criteria for MAiD-MD do not take into consideration key features of certain mental disorders, like fluctuating suicidal ideations and behaviours [6]. One article deplored that some healthcare professionals believe that patients who have the physical capacity to commit suicide should do so rather than resort to MAiD-MD and to the resources this practice mobilizes [31, 48]. Several authors express a profound unease with this idea, knowing that letting the patient take matters into their own hands can lead to a violent and isolated death or to an even more painful situation in the event of a failed suicide attempt [30, 41, 63]. In this sense, Berghmans et al. (2013) express that people living with mental disorders generally do not have the means to end their life with dignity without the help of healthcare professionals [71]. An alternative to MAiD-MD could be voluntarily stopping eating and drinking, but MAiD-MD is perceived as being more humane [30, 68]. The fate of patients being refused MAiD-MD [83], as well as the impact of MAiD-MD on loved ones [55] and on other people living with mental disorders [59, 68], are noted as morally concerning and remain understudied.

### Identified needs and proposed solutions to issues with MAiD-MD practices

The literature is replete with questions to address in relation to MAiD-MD, including the feeling of being a burden [20, 69]; safeguards to implement or avoid [30, 56, 69]; factors reducing the voluntariness of MAiD-MD requests [46]; the relevance of adopting a more holistic consideration of certain eligibility criteria, by better considering the quality of life and the needs of patients [48]; the importance of developing resources oriented towards aid in living, etc. A need for training [78], awareness-raising [27], and guidelines [78] is apparent, as well as a need to develop more support resources to help all stakeholders through the challenging situations and discussions that can arise in the context of MAiD-MD [64, 70, 84].

## Discussion

### Critical analysis of the literature on MAiD-MD

The thematic analysis of available publications has made it possible to identify various moral concerns and possible remediations, which are related to how MAiD-MD is introduced in five contexts of emergence. The societal context can influence some people towards MAiD-MD because of stigmatizing experiences or difficult living conditions. The healthcare system poses certain barriers to living with a mental disorder, both through limited access to insufficient resources, as well as gaps in knowledge, medical training and suicide prevention. The continuum of care is challenged with respect to quality of care, response to needs, goals of care, hope and the therapeutic relationship. Those who wish to discuss the MAiD-MD option encounter relational and communication challenges. MAiD-MD practices are complicated by different types of uncertainty, both in the assessment of MAiD-MD applications and in the quality and repercussions of the processes. The assessment of MAiD-MD applications, which is part of the context of MAiD-MD practices, is the most recurrent source of concern but addressing the various moral concerns that emerge upstream of MAiD-MD (e.g., moral concerns emerging in the other four contexts identified) could indirectly facilitate the assessment of requests.

Three general observations emerge from this review. First, based on the sample of literature included in this review, we observe a lack of qualitative empirical studies reporting the perspectives of people living with mental disorders and their relatives. Second, we note points of convergence in the literature: certain morally concerning constructs transcend the contexts of emergence and thus seem particularly worrying. Third, taking a critical look at the possible solutions, we note that they are mainly oriented towards what should be done to remedy the concerns, without proposing how to do so.

#### Lack of qualitative empirical studies

Only 4 of the 74 articles included are qualitative empirical studies, and only one directly relates to the moral concerns of people living with mental disorders: the others deal with the perspectives of clinicians. The remaining 70 articles are contributions from clinicians or from academics. Among these articles, some (e.g., comments written by clinicians) relate the moral concerns of patients or relatives, but this is done indirectly, via the interpretation of the authors. Based on the consulted literature, the perspectives of patients and their relatives thus seem poorly documented, and their being often reported by others can influence how they are communicated. However, a growing body of empirical qualitative research on medical assistance in dying for people with mental disorders has emerged in recent years [85–87]. Some relevant articles, which would have emerged with a search strategy focusing solely on medical assistance in dying and mental disorders, may have been overlooked by the search strategy. It may be the case because when someone talks about their experience or shares their perspective on a sensitive subject, the moral dimensions of these experiences are not always made explicit, despite being implicitly present. Further enrichment of this body of empirical qualitative literature by clarifying the moral aspects of lived experience would increase our understanding of the discomfort experienced by those involved in medical assistance in dying for mental health reasons.

#### Points of convergence in the literature

The classification of moral concerns by contexts of emergence has enabled us to identify that four morally concerning constructs recur in different contexts. The fact that a given problem occurs in several ways, particularly in different contexts, suggests that it is widespread. We believe that the recurrence of these problematic constructs suggests the importance of addressing them as a priority. The first is stigma, experienced in society but also in care experiences. The second relates to the notion of burden: MAiD-MD entails a certain level of burden, particularly for the healthcare system; for clinicians feeling the weight of their professional responsibility in the context of care and MAiD-MD; for loved ones and clinicians feeling helpless in the face of persistent suffering. The third construct relates to relational and communication challenges arising in the care trajectory, upstream and downstream of requests for MAiD-MD: biases, uneasiness and judgments can show through in communication, and some people can then feel distress. The fourth construct concerns the factors influencing the request for MAiD-MD: environmental factors, other people and the mental disorder itself can alter the autonomous nature of the request by influencing it unduly. Although not all requests for MAiD-MD are marked by these influences, it is important to be attentive to them in order to prevent requests from being accepted when other options could have better met the needs of the applicants.

#### Critical look at moral concerns and proposed solutions

Given the large number of moral concerns documented, it is essential, on the one hand, to prioritize certain concerns that seem more important or more recurrent, as we have undertaken to do by identifying specific points of convergence in the literature. On the other hand, we need to consider the source of these moral concerns. Considering that moral concerns are lived by people and thus form part of their experience, we recognize that all moral concerns can be genuinely worrying for the people who experience them but that some may have to be addressed at source. For example, some moral concerns may stem from misunderstanding the laws or practices of medical assistance in dying. They emerge from a lack of information and can therefore be addressed more quickly than others, notably by explaining the laws, practices, and implications more clearly.

Moreover, in the current literature, the proposed solutions are generally oriented towards “what to do” to remedy a given moral concern – ideas for addressing the problems are proposed – without explaining “how” to remedy the problem in order to promote the well-being of those concerned. For example, if communication issues are interfering with the clinician-patient relationship, the importance of open communication will be emphasized, as will the importance of cultivating the therapeutic relationship, but the recommendations will generally not go as far as to suggest “how to do this”. However, while “how to” ideas are not well documented in the scientific literature, some in-depth reports emerging from the grey literature, notably in Canada, go further, accompanying their “what to do” recommendations with a few pointers to guide “how” this might be implemented locally. For a given moral concern, it would seem promising to develop, with the concerned stakeholders, tools or resources that are adapted to each emergence context. For example, stigma could be addressed at the societal level through awareness campains, and at the healthcare level through training tailored to the nuances and subtleties of stigma and self-stigma. While stigma is not specific to MAiD-MD, it can significantly affect how MAiD-MD requests are responded to and assessed and how people living with mental disorders experience the process. The prejudice that leads some people to consider anyone living with a mental disorder de facto unfit to make a legitimate request for medical assistance in dying can also lead to systematic refusals of those requests. Thus, different reversible factors that may unduly influence the request for MAiD-MD could be tackled before considering going forward with MAiD-MD. In order to limit difficult relational experiences, tools could be developed to improve the recognition of biases: this type of introspection exercise could help communication in care, as well as in the reception and assessment of MAiD-MD applications. Facilitating difficult discussions could also be beneficial in easing the burden felt by different stakeholders. Finally, although certain moral concerns relating to MAiD-MD seem particular to the context of mental disorders, several of them could emerge in the context of MAiD more broadly, and thus, the solutions to answer them may have already been developed in physical MAiD, and could therefore be applied in the context of mental disorders.

#### Limits

A first limitation of this review is the methodological choice of excluding the grey literature, which has inevitably led us not to consider relevant reflection work on MAiD-MD such as the recent Final Report of Canada’s Expert Panel on MAiD and Mental Illness [88]. A second limitation is that considering that the moral dimensions of human experience are often implicit, the methodological choice of limiting the literature search to writings containing keywords related to ethics or morality in addition to keywords related to MAiD and mental disorders may have excluded some relevant articles. A third limitation is that given the qualitative orientation of this review, the search was restricted to publications with a certain depth of qualitative analysis. Some relevant articles, such as quantitative empirical research, could thus have been discarded. A fourth limitation is that certain disorders, such as neurocognitive disorders, autism spectrum disorders and intellectual disabilities, were excluded for the sake of not grouping overly heterogeneous conditions together, but certain identified moral concerns identified in this review could still concern those groups. We excluded the articles on those disorders in order to delimitate clearly the literature review. Also, we consider that the potential similarities in the experienced moral concerns should be demonstrated, not assumed.

## Conclusion

MAiD-MD raises fundamental ethical concerns that need to be addressed. This literature review is one of the first to look at the moral concerns related to MAiD-MD and possible solutions to address them. We hope that this advancement will guide the development of resources, interventions or support tools aimed at improving the experiences of people living with mental disorders, their loved ones and healthcare professionals, who experience these concerns.

### Electronic supplementary material

Below is the link to the electronic supplementary material.

Supplementary Material 1

## Data Availability

Not applicable.

## Footnotes

1 The context of discussions on the option of MAiD-MD refers to the period of reflection surrounding this option as well as the request itself. A person may consider requesting MAiD-MD and wish to talk about it with those around them and their clinicians, then decide to go ahead or not. Consideration of MAiD-MD may include a request for MAiD-MD, which will then be explored through discussions before moving on to assessment (which is part of the context of MAiD-MD practices).

## Abbreviations

MAiD-MD: Medical Assistance in Dying (in a context of) Mental Disorder
MeSH: Medical Subject Headings
